# Magnitude of adverse drug reaction and associated factors among HIV-infected adults on antiretroviral therapy in Hiwot Fana specialized university hospital, eastern Ethiopia

**DOI:** 10.11604/pamj.2016.24.255.8356

**Published:** 2016-07-20

**Authors:** Fitsum Weldegebreal, Habtamu Mitiku, Zelalem Teklemariam

**Affiliations:** 1 Haramaya University, College of Health and Medical Sciences, Harar, Ethiopia

**Keywords:** Adverse drug reaction, Human Immuno deficiency virus, adults

## Abstract

**Introduction:**

Human immunodefiecency virus infected patients did not adhere correctly to their Antiretroviral Therapy because of the drugs adverse effects. Thus, continuous evaluation of the adverse effect of Antiretroviral Therapy will help to make more effective treatment. The aim of this study was to assess the prevalence of Adverse Drug Reaction and associated factors on Antiretroviral Therapy among Human immunodefiecency virus infected Adults at Hiwot Fana Specialized University Hospital, Eastern Ethiopia.

**Methods:**

A Hospital based retrospective study was conducted among 358 of adult patients clinical records on antiretroviral Therapy from April1 to June30, 2014.

**Results:**

The overall prevalence of Adverse Drug Reaction among Human immunodefiecency virus infected patients on antiretroviral Therapy was 17.0%. Of reported Adverse Drug Reaction, 80.3%, 18% and 1.7% occurred in patients on Stavudine, Zidovudine and Tenofovir based regimens respectively. The common Adverse Drug Reaction were lipodystrophy (fat change) (49.2%), numbness/tingling (27.9%), peripheral neuropathy (18%) and (8.2%) anaemia (8.2%). Patients on Stavudine containing regimens were more likely to develop Adverse Drug Reaction compared to Zidovudine (AOR = 0.212, 95% CI 0.167, 0.914, p<0.001) and Tenofovir (AOR=0.451, 95% CI 0.532, 0.948, p<0.001).

**Conclusion:**

The overall prevalence of Adverse Drug Reaction among Human immunodefiecency virus infected patients in this study was 17% and more common on those patients taking Stavudine based regimen. Lipodystrophy and peripheral neuropathy were significantly associated with stavudine-based regimens, while anaemia was significantly associated with zidovudine based regimens. Thus regular clinical and laboratory monitoring of patients on Antiretroviral Therapy should be strengthened.

## Introduction

Antiretroviral Therapy (ART) is the cornerstone of management of patients infected with Human immune deficiency virus (HIV). The initiation ART has resulted in significant decreases in HIV-related morbidity and mortality in both the developed and developing countries [1–3]. It is also increases the length and quality of life and productivity of patients by improving survival and decreasing the incidence of opportunistic infections through reduction of the viral load and increasing the level of CD4 T cells [4]. Worldwide, it is estimated that between 250,000 and 350,000 deaths were averted in 2005 as a result of increased ART treatment accesses [5]. In Ethiopia, fee based ART program began in July 2003. By March 2005, Global Fund funded free ART service started in at least one site in all the regions [6, 7]. The success of the ART is highly dependent on willingness of HIV infected patients to strictly adhere to the regimens [8, 9]. However these drugs are associated with adverse effects. Adverse Drug Reactions (ADRs) are responses to a medicine which is noxious and unintended, and which occurs at doses normally used in human? [3]. It has been pointed as one of the main reason for discontinuation, switch and non-adherence to ART [10]. Up to 25% of patients discontinue their initial ART regimen within the first 8 months of therapy because toxic effect of ART [11]. The prevalence of ADRs is variably reported from different studies which were 66.7% in USA, 71.11% in India 71.11%, 4.7% in South Africa, and 88% in Ethiopia [12, 13]. There was limited information about the magnitude and associated factors of ADRs among patients on ART in Ethiopia [3]. There is also no published report about the magnitude and associated factors of ADRs in Harari National Regional State. Therefore, this study assessed the magnitude of ADRs and associated factors on ART among HIV-infected adults at Hiwot Fana Specialized University Hospital, Eastern Ethiopia.

## Methods

**Study area and population:** Harari People National Regional State is located in the Eastern part of Ethiopia which is 515 kms away from the capital city- Addis Ababa. It had a projected total population of 203,438 in 2010. It has 36 kebeles (17 rural and 19 urban), which are the lowest administrative clusters in the region. The health service coverage of the region, which is calculated based on the number of health institutions giving health services per population in the region, is estimated to be about 100%. There were 6 Hospitals and 8 health centers in the region [14]. ART program was launched on March 26, 2006 in the region. Three thousand two hundred nineteen (3290) patients had been initiated ART between March 2006 and June26, 2014. Among the total ART patients, about 1960 (69.6%) were on treatment currently, but 487 (14.8%), 343 (10.4%) and 250 (7.6%) ART patients were transferred out, dropped and died respectively. Currently, ART services are being given to HIV/AIDS patients in two public hospitals in the region. This study was conducted in Hiwot Fana Specialized University Hospital ART unit, which is one of the hospitals in the regional state. All HIV infected patients of ART clinical cards in the region were the source population. Those ART clinical records of HIV infected patients, whose age is 18 years and above, in Hiwot Fana Specialized university hospital ART clinic were study population.

**Study design and period**: A Hospital based retrospective review of ART clinical records were conducted among patients who initiated ART between March26, 2006 and March 26, 2014.

**Sample size estimation and sampling techniques**: The sample size was determined by using single proportion formula by setting 95% confidence interval, P= 0.303 of patients taking ART develop ADRs [15], margin of error 0.05. The final sample size was 325, including 10% missing or incomplete record rate. The final sample size was 358. The final study participants were selected by systematic random sampling using the ART registration book from the ART unit of the study hospital as sampling frame.

**Data collection:** Data were collected using structured questionnaire which was adopted from routine standard ART monitoring card. Information on patient's details like socio demographic characteristics, WHO clinical staging of the disease at the starting of ART, duration of treatment, drug details, types, severity and outcome of ADRs of reported by patients during clinical examination at time coming to ART unit for their appointment visit were collected by clinical nurses who are working at the hospital ART Clinic. Haematological and metabolic ADRs, which could be done by laboratory examination, were not collected from ART clinical records. The data collection format was checked on daily bases by investigators and supervisors for its completeness and consistency with the patient's clinical records of the hospitals.

**Data entry and analysis:** The collected data were double entered, cleaned and analyzed by using SPSS soft-ware Version16. The finding of this study was presented by using mean, standard deviation and simple frequencies tables. The overall prevalence of ADRs was determined as the proportion of individuals who developed the different ADRs by clinical examination. Odd ratio with 95% confidence interval was used to describe association between the selected study variables (i.e. Outcome and independent variables). Univariate and multivariable logistic regression analysis was performed to explore independent variables that were predictors of adverse drug reactions (ADRs). The criterion for significance was set at P < 0.005. The severity of ADRs were categorized from grade I to IV based on criteria used the WHO severity grading [16].

**Ethical consideration:** The study protocol was approved by Institutional Health Research and Ethical Review Committee of College of Health and Medical Sciences, Haramaya University. And the patients’ clinical records were reviewed anonymously and all information obtained from clinical records was kept confidential.

## Results

**Characteristics of the study subjects:** A total of 358 HIV/AIDS patient ART clinical records were reviewed in this study. The mean age of patients was 34 (SD + 9.8) years, ranging from 18-71 years. Most of the patients were on the age group of 30-39 years (41.4%), female (68.4%), Urban (83%) and elementary school (44.7%). One hundred fifty (41.9%) of the study participants were at WHO clinical stage III. With regards to functional status, most (65.9%) of them were working. Majority (74.6%) of the study participants were on the first line ART regimen. D4T (30)/3TC/ NVP (33.2%) were the most commonly prescribed ART regimen at their initial time initiation of ART. The most frequently used drug other than ART among the study participants were Cotrimoxazole (75.7%) and Isonizide (INH) (14.5%) (Table 1).

**Table 1:** Characteristics of study participant (n=358) at Hiwot Fana Specialized University Hospital ART Clinic, Harar, Eastern Ethiopia, 2014

Variables		Number (%)
**Sex**	Male	113 (31.6)
	Female	245 (68.4)
**Marital status**	Single	58(16.2)
	Married	160(44.7)
	Divorced	72(20.1)
	Widowed	68(19.0)
**Educational status**	Illiterate	58(16.2)
	Primary school(1-8 grade)	160(44.7)
	Secondary(9-12 grade)	108(30.2)
	College/University	32(8.9)
**Residence**	Urban	297(83.0)
	Rural	61(17.0)
**Age group**	18-29	117(32.7)
	30-39	148(41.4)
	40-49	66(18.4)
	>=50	27(7.5)
**WHO clinical stage**	I	70(19.6)
	II	95(26.5)
	III	150(41.9)
	IV	43(12.0)
**Functional status**	Work	236(65.9)
	Ambulatory	99(27.7)
	Bed ridden	23(6.4)
**drug of initial regimen at Initiation ART**	D4T (30)/3TC/ NVP	119(33.2)
	D4T (40)/3TC/NVP	17(4.7)
	D4T (30)/3TC/EFV	31(8.7)
	D4T (40)/3TC/EFV	7(2.0)
	AZT/3TC/ NVP	61(17.0)
	AZT/3TC/EFV	32(8.9)
	TDF/3TC/NVP	30(8.4)
	TDF/3TC/EFV	60(16.8)
	TDF/ddl/LPV/R	1(0.3)
**Drug used other than ART**	Cotrimoxazole	
	Yes	271(75.7)
	No	87(24.3)
	INH	
	Yes	52(14.5)
	No	306(85.5)
	Anti-Tuberculosis drug	
	Yes	23(6.4)
	No	335(93.7)

NFV- nelfinavir; AZT- zidovudine; 3TC - lamivudine; EFV - efavirenz; D4T-staviduine INH-Isonizaide

**Antiretroviral treatment (ART) Drug regimen and follow up status of HIV/AIDS patients**: At the time of study, 33.2% of the study participants were still on their initial regimen whereas 38% of them had changed one or two drugs during their follow up. Currently (during the study period), most (69.7%) of the HIV/AIDS patients on ART were in the first line regimens. The main reasons for changing initial regimen of ART drugs were new drug availability (45.6%) and adverse drug reaction (41.2%) followed by Immunological failure 10(7.4%), due to new TB 9(6.6%), risk of pregnancy, clinical failure, virological failure, immunological failure and clinical failure, and immunological failure and viral failure each accounts 1(1%). Two hundred fifty eight (72.1%) were still on ART follow up in the hospital ART clinic whereas 48(13.4%), 29(8.15) and 24(6.7%) of the study participants were transferred out to other ART clinic site, died and interrupted their follow up respectively. ART treatment adherence rate among study participants was also reviewed from participant's card. About (5%) had poor adherence. The main reasons for poor adherence were lost / ran out of pills 10(55.6%) and delivery/ travel problems 4(22.2%), followed by nutritional problem 1(5.6%), taking alcohol1 (5.6%), too ill 1(5.6%) and combination of stigma 1(5.6%).

**Magnitude, clinical symptoms and severity of adverse drug reactions (ADRs):** The overall prevalence of ADRs among these study participants was 17.0%. The most frequently identified ADRs clinical symptoms were lipidtropy (Fat change) (44.3%) and Numbness/tingling (4.9%). Most (80.3%) of the patients had grade III ADR. The main management/measure taken to resolve ADRs were one drug change (62.3%), reassurance (18%) and regimen change (14.8%). The majority 27/61 (44.3%) of the study participants with ADRs were died who were on grade III. In addition, most of the study participants, who developed ADRs, were WHO stage III (47.5%) (Table 2). Nausea, numbness/tingling, fatigue, headache, jaundice, fat change, both numbness/tingling and fat changes were mostly reported in those study participants who were using D4T based drugs. While anemia was highly reported in those study participants who were using AZT based drugs (Table 3).

**Table 2:** Prevalence, clinical symptoms, severity, measures taken and outcomes of ADRs among HIV/AIDS patients on ART at Hiwot Fana Specialized University Hospital ART Clinic, Harar, Eastern Ethiopia, 2014

Variables			Percent (%)
**Patients developed ADRs**	Yes		17.0
	No		83.0
**Clinical symptoms of ADRs**	Nausea		4.9
	Fatigue		1.6
	Diarrhea		3.3
	Headache		1.6
	Numbness/tingling		23
	Rash		6.6
	Anemia		8.2
	Jaundice		1.6
	Fat changes		44.3
	Numbness/tingling and fat changes		4.9
**Measure taken for ADRs**	Reassurance		18.0
	one drug change		62.3
	regimen changed		14.8
	all drug stopped		1.6
	Supportive Rx and one drug change		3.3
**Outcome of ADRs**	Death		47.5
	Recover with sequelae		27.9
	Recover without sequelae		24.6
**Severity**	Grade I	Death	18.0
		Recover without sequelae	
	Grade III	Death	80.3
		Recover with sequelae	
		Recover without sequelae	
	Grade IV	Death	1.7
**WHO clinical stage**	I		16.4
	II		29.5
	III		47.5
	IV		6.6

**Table 3:** Distribution of ADRs by ART regimen based prescribed at first initiation treatment among HIV/AIDS patientson ART at Hiwot Fana Specialized University Hospital ART Clinic, Harar, Eastern Ethiopia, 2014

ADRs	Drug of regimen base			Total	P -value
	D4T	AZT	TDF		
Nausea	66.7%	33.3%	0.0%	100.0%	0.02
Diarrhea	100.0%	0.0%	0.0%	100.0%	
Fatigue	100.0%	0.0%	0.0%	100.0%	
Headache	100.0%	0.0%	0.0%	100.0%	
Numbness/tingling	92.9%	7.1%	0.0%	100.0%	
Rash	0.0%	75.0%	25.0%	100.0%	
Anemia	40.0%	60.0%	0.0%	100.0%	
Jaundice	0.0%	100.0%	0.0%	100.0%	
Lipodystrophy/ Fat changes	92.6%	7.4%	0.0%	100.0%	
Numbness/tingling and fat changes	100.0%	0.0%	0.0%	100.0%	

**Factors associated with adverse drug reactions:** The prevalence of ADRs was higher in those study participants on D4T based ART drug regimen especially D4T/3TC/EFV. This was significantly different when compared to study participants in other treatment regimens (P<0.005). The prevalence of ADRs was higher in those study participants who were more than 50 years of age, in females, ambulatories, in WHO stage III, with initial CD4 count 201-300 cells/µl and grade III severity level. But the difference was not statistically significant when comparisons were made within each category member (P>0.05). Further analysis with univariate and multivariate was done. In both univariate and multivariate analysis all ART drug users were less likely to develop ADRs as compared to D4T/3TC/ NVP users (p<0.05). It was compared based on the base ART drugs; AZT and TDF based ART drug regimen base users were less likely to develop ADRs when compared to D4T base users (Table 4).

**Table 4:** Factors associated with ADRs among HIV/AIDS patients on ART at Hiwot Fana Specialized University Hospital ART Clinic, Harar, Eastern Ethiopia, 2014

Variables		Patient developed ADR		Crude OR	95% Confidence Interval		p-value	Adjusted OR	95% Confidence Interval		p-value
		Yes	No		Lower Bound	Upper Bound			Lower Bound	Upper Bound	
Drug base of initial regime	D4T	28.2%	71.8%	1			**0.003**	1			**0.001**
	AZT	11.8%	88.2%	0.922	**0.436**	**0.988**		**0.212**	0.167	0.914	
	TDF	1.1%‘	98.9%	0.280	**0.083**	**0.845**		**0.451**	0.532	0.948	
Age	18-29	10.3%	89.7%	1				**1**			**0.173**
	30-39	17.6%	82.4%	**2.704**	0.902	6.573	0.061	1.781	1.342	7.015	
	40-49	10.6%	89.4%	**2.758**	1.901	7.021		1.644	2.172	5.740	
	>=50	22.2%	7.8%	**1.941**	**1.561**	**8.201**		**3.21**	**2.736**	**8.214**	
Drug of initial regimen	D4T /3TC/ NVP	24.3%	75.7%	1				1			**0.01**
	D4T/3TC/EFV	42.1%	57.9%	0.657	0.223	0.987	0.002	0.494	1.644	2.172	
	AZT/3TC/ NVP	9.8%	90.2%	0.279	0.112	0.720	0.005	0.768	2.740	2.736	
	AZT/3TC/EFV	15.6%	84.4%	0.372	0.173	0.817	0.040	0.836	1.970	2.578	
	TDF/3TC/NVP	3.3%	96.7%	0.173	0.045	0.909	0.028	0.874	1.510	2. 989	
	TDF/3TC/EFV	0.0%	100%	0.679	0.382	4.789	0.009	0.947	1.086	2.237	
	TDF/ddI/LPV/R	0.0%	100%	0.781	1.210	3.763	0.039	0.957	2.532	2.948	
Sex	Male	12.4%	87.6%	1			**0.071**	1			**0.073**
	Female	19.2%	80.8%	**2.734**	2.651	8.125		**3.251**	1.642	8.261	

## Discussion

In this study, 17% of the HIV/AIDS patients on ART were developed atleast one ADRs. This is similar to finding of study conducted in India (17.5%) and Cameroon (19.5%) [17, 18] but it was lower than the finding of study in Gondar University Hospital (89.8%), Ethiopia [19] and 37% in south Africa [20]. This difference may be due to difference in types ADRs in different studies. In this study 9 ADRs symptoms were reported. But, more than 14 ADRs symptoms were reported from a cross sectional study conducted in Gondar University Hospital study [19]. The other reason might be due to all laboratory tests were not fully performed and not well documented as per ART monitoring guideline in this study. Thus haematological and metabolic adverse reaction were not detected. This can underestimate the magnitude of ADRs. The prevalence of ADRs was higher in those study participants on Stavudine-based ART drug regimen. This finding was similar to a study conducted in Cameroon [18]. But contrary to previous reports from Nigeria, India and South Africa which found ADR was less reported from patients on Stavudine-based regimens compared to Zidovudine -based regimens [20–22]. In this study, the most commonly encountered ADR was lipodystrophy. The main clinical features such as lipodystrophy lipoatrophy and lipohypertrophy were reported from HIV/AIDs patients on ART as main ADRs [23, 24]. The occurrence of lipodystrophy in the present study was higher compared to other studies. The prevalence of lipodystrophy varies considerably across studies by 3.5%- 34.2% in Kenya, Cameroon, Botswana and Rwanda [18, 25–27]. This variability might be due to multiple risk factors for lipodystrophy such as genetic factors, low body weight before therapy, raised C-peptides and triglyceride concentrations after about 1 year, use of dual Protease inhibitor (PI) combination ritonavir-saquinavir and use of nucleotide analogue, stavudine [15, 23]. Majority of lipodystrophy occurred in those study participants who were on a Stavudine based regimen. Lipodystrophy was also a reason for treatment change in nearly 1 of every 2 patients with ADRs. This was most all similar to the study conducted in Rwandan and Cameroonian HIV patients on stavudine who were more likely to develop lipodystrophy compared to those on Zidovudine. Lipodystrophy was the reason for treatment change in 1 of every 4 patients with ADRs [18, 27].

The other common ADR was peripheral neuropathy (18%). This was similar to the study carried out in Cameroon (21.2%) and Kenya (20.7%) [18, 25]. However, it was lower than the findings in Botswana (23%) [26]. These differences might be explained by the fact that the Botswana study was a randomized clinical trial in which all patients on ART were intensely screened to search for ADRs [26]. It might also due difference in reporting ADRs by HIV/AIDS patients from different parts world. The majority of peripheral neuropathy occurred in those patients on Stavudine based regimen. This was also reported in studies conducted in Cameroon and Nigeria. Even though, in both studies majority of the study participants were on AZT based regimens [21, 18]. For Hematological ADRs, anaemia was only recorded, which accounts for 8.2% of ADRs, in this study. This finding was similar to the study conducted in Senegal (7.8%) [26]. But it was higher than the studies reported in Ethiopia- from Tikur Anbesa hospital (4.8%), Cameroon (3.8%) and Thailand (3.8%) [18, 28, 29]. These differences might reflect the variation in relative use of AZT containing regimens in different settings. In this study, the majority (60%) of anaemia was reported from patients taking AZT based regimen. This was in agreement with the study conducted in Cameroon and Thailand [18, 29]. Data were collected from clinical record which record mostly clinical reports. Laboratory tests were not performed for most of the patients, even not well documented as per ART monitoring guideline. So, haematological and metabolic ADRs were not collected from the record. This might underestimated the magnitude of ADRs in this study.

## Conclusion

The prevalence of ADRs was 17% and more common on those patients taking Stavudine based regimen. Lipodystrophy, peripheral neuropathy and anaemia were the common ADRs. Lipodystrophy and peripheral neuropathy were significantly associated with stavudine-based regimens, while anaemia was significantly associated with zidovudine based regimens. Therefore this study recommends, regular clinical and laboratory monitoring of patients on ART should be strengthened before and after initiation of ART according to guideline of institution particularly for stavudine and zidovudine based regimens. It also recommends further large scale study to assess the magnitude and associated factors ADRs by using clinical and laboratory examination.

### What is known about this topic

- The prevalence of ADRs was more common on those patients taking Zidovudine -based regimens;
- Lipodystrophy and peripheral neuropathy were significantly associated with stavudine-based a regimen;
- Anemia was significantly associated with zidovudine based regimens.

### What this study adds

- The prevalence of ADRs was more common on those patients taking Stavudine based regimen;
- Lipodystrophy was the common ADRs.

## References

[1] Bonnet F, Morlat P, Chêne G, Mercié P, Neau D, Chossat I (2002). Causes of death among HIV-infected patients in the era of highly active antiretroviral therapy, Bordeaux, France, 1998-1999. HIV Med.

[2] Etard J, Ndiaye I, Thierry-Mieg M, Guèye N, Guèye P, Lanièce I (2006). Mortality and causes of death in adults receiving highly active antiretroviral therapy in Senegal: a 7-year cohort study. AIDS Lond Engl.

[3] Teklay G, Legesse B, Legesse M (2013). Adverse Effects and Regimen Switch among Patients on Antiretroviral Treatment in a Resource Limited Setting in Ethiopia. J Pharmacovigilance.

[4] Hogg R, Yip B, Kully C, Craib p, O'Shaughnessy v, Schechter T, Montaner G (1999). Improved survival among HIV-infected patients after initiation of triple-drug anti-retroviral regimens. CMAJ.

[5] WHO/UNAIDS (2006). Report on the global AIDS epidemic, treatment and care.

[6] FMOH and HAPCO (1998). ART update in Ethiopia.

[7] Hogan D, Salomon J (2005). Prevention and treatment of HIV/AIDS in resource limited settings. Bulletin of WHO, Geneva.

[8] Ammassari A, PaolaTrotta M, Mul R (2005). Correlates and Predictors of Adherence to HAART. JAIDS.

[9] FMOH (2006). Disease Prevention and Control Department, AIDS in Ethiopia.

[10] Shah I (2006). Adverse effects of antiretroviral therapy in HIV-1 infected children. J Trop Pediatr.

[11] Montessori V (2004). Adverse effects of antiretroviral therapy for HIV infection. Canadian Medical Association Journal.

[12] Brinkman K, Smeitink JA, Romijn JA, Reiss P (1999). Mitochondrial toxicity induced by nucleoside-analogue reverse-transcriptase inhibitors is a key factor in the pathogenesis of antiretroviral-therapy-related lipodystrophy. Lancet.

[13] Lucas GM, Chaisson RE, Moore RD (1999). Highly active antiretroviral therapy in large urban clinic: risk factors for neurologic failure and adverse drug reaction. Ann Intern Med.

[14] Federal Democratic Republic of Ethiopia-Ministry of Health (2010). Harari region Health Sector Development Program IV (2010/11-2014/15) plan Zero draft, Harar.

[15] Habtegiorgis A, Gebreyesus S, Mulugeta E (2003). Evaluation of antiretroviral treatment in two private medical centers in Addis Ababa. Ethiop Med J.

[16] WHO (2006). Antiretroviral therapy for HIV infection in adults and adolescents: recommendations for a public health approach.

[17] Modayil R, Harugeri A, Parthasarathi G, Ramesh M, Prasad R, Naik V, Giriyapura V (2010). Adverse drug reactions to antiretroviral therapy (ART): an experience of spontaneous reporting and intensive monitoring from ART centre in India. Pharmacoepidemiology and Drug Safety.

[18] Luma HN, Doualla MS, Choukem SP, Temfack1 E, Ashuntantang G, Joko1 HA, Koulla-Shiro S (2012). Adverse drug reactions of Highly Active Antiretroviral Therapy (HAART) in HIV infected patients at the General Hospital, Douala, Cameroon: a cross sectional study. Pan African Medical Journal.

[19] Tadesse TW, Mekonnen BA, Tesfaye HW, Tadesse TY (2014). Self-reported adverse drug reactions and their influence on highly active antiretroviral therapy in HIV infected patients: a cross sectional study. BMC Pharmacology and Toxicology.

[20] Masenyetse L, Manda S, Mwambi H (2015). An assessment of adverse drug reactions among HIV positive patients receiving antiretroviral treatment in South Africa. AIDS Research and Therapy.

[21] Agu KA, Oparah AC (2013). Adverse drug reactions to antiretroviral therapy: Results from spontaneous reporting system in Nigeria. Perspect Clin Res.

[22] Sharma A, Vora R, Modi M (2008). Adverse effects of antiretroviral treatment. Indian J Dermatol Venereol Leprol.

[23] Marfatia Y, Smita M (2005). Adverse Drug Reactions (ADR) due to Anti-retrovirals (ARV): Issues and Challenges. Indian J Sex Transm Dis.

[24] WHO (2012). Patient evaluation and antiretroviral treatment for adults and adolescents, clinical Protocol for the WHO European Region (2012 revision).

[25] Chang KH, Kim JM, Song YG, Hong SK, Lee HC, Lim SK (2002). Does race protect an oriental population from developing lipodystrophy in HIV-infected individuals on HAART?. The Journal of Infection.

[26] Wester CW (2007). Higher-than-expected rates of lactic acidosis among highly active antiretroviral therapy-treated women in Botswana: preliminary results from a large randomized clinical trial. Journal of Acquired Immune Deficiency Syndromes.

[27] Van Griensven J, De Naeyer L, Mushi T, Ubarijoro S, Gashumba D, Gazille C, Zachariah R (2007). High prevalence of lipoatrophy among patients on stavudine-containing first-line antiretroviral therapy regimens in Rwanda. Transactions of the Royal Society of Tropical Medicine and Hygiene.

[28] Abdissa SG, Fekade D, Feleke Y, Seboxa T, Diro E (2012). Adverse drug reactions associated with antiretroviral treatment among adult Ethiopian patients in a tertiary hospital. Ethiop Med J.

[29] Nuesch R, Srasuebku P, Ananworanich J, Ruxrungtham K, Phanuphak P, Duncombe C (2006). Monitoring the toxicity of antiretroviral therapy in resource limited settings: a prospective clinical trial cohort in Thailand. Journal of Antimicrobial Chemotherapy.

